# Supplementary figures and images for: Applying machine learning to predict stunting in children under 5 years old based on water, sanitation and hygiene behaviors and infrastructure
Source: PLoS One. 2026 Mar 5;21(3):e0343796. doi: 10.1371/journal.pone.0343796 (PMC12962480; doi:10.1371/journal.pone.0343796)

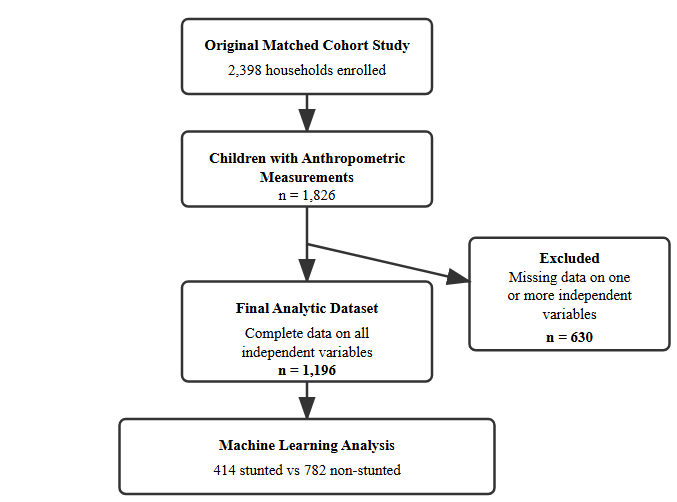

Supplement: S1 Fig — (TIF) [file pone.0343796.s001.tif]
