# Supplementary material for: Applying machine learning to predict stunting in children under 5 years old based on water, sanitation and hygiene behaviors and infrastructure
Source: PLoS One. 2026 Mar 5;21(3):e0343796. doi: 10.1371/journal.pone.0343796 (PMC12962480; doi:10.1371/journal.pone.0343796)
Supplement: Attachment — Submitted filename: Response to Reviewers.docx [file pone.0343796.s003.docx]

**Response to Reviewers**

Dear Dr. Ashish Wasudeo Khobragade,

Thank you for your feedback on our manuscript titled "Applying machine learning to predict stunting in children under 5 years old based on water, sanitation and hygiene behaviors and infrastructure" (PONE-D-24-38002). We appreciate the opportunity to revise our work according to your suggestions and those of the reviewers. We have carefully addressed all comments and concerns in this revision. Below, we provide point-by-point responses to each comment.

**Journal Requirements:**

| **Comment** | **Response** |
| --- | --- |
| 1. Please ensure that your manuscript meets PLOS ONE's style requirements, including those for file naming. | We revised our manuscript to ensure it meets PLOS ONE's style requirements. We also followed the file naming guidelines as specified by the journal. |
| 2. Please note that PLOS ONE has specific guidelines on code sharing for submissions in which author-generated code underpins the findings in the manuscript. | As requested, we agree to make author-generated code available upon publication of this work. |
| 3. Amended Funding Statement | Amended Funding Statement included with this letter and cover letter. |
| 4. Remove funding-related text from the Acknowledgments section | We removed all funding-related text from the Acknowledgments section of the manuscript. Amended Acknowledgements included with this letter and cover letter. |
| 5. In the online submission form, you indicated that [The datasets used and/or analyzed during the current study are available from the corresponding author on reasonable request]. All PLOS journals now require all data underlying the findings described in their manuscript to be freely available to other researchers. | The data underlying the findings described in this manuscript have been uploaded to FigShare. They can be accessed via the following private link, which will be updated to a publicly available DOI upon acceptance: <https://figshare.com/s/5678f9122fcc78739a2d> |

**Additional Editor Comments:**

| **Comment** | **Response** |
| --- | --- |
| In this study, the XGBoost model is used to predict stunting. The authors mention moderate performance of other models in the limitations. What are the limitations of using the XGBoost model? What is the effect of overfitting and different data sets on the results? | Thank you for this important question. We have expanded our discussion of XGBoost limitations in the revised manuscript to reflect the below in the “Discussions” section:  While XGBoost demonstrated superior predictive performance in our study, it has limitations. Despite our use of k-fold cross-validation and hyperparameter tuning, XGBoost models remain susceptible to overfitting. The model's performance is also dependent on the specific characteristics of our dataset, and these results may not generalize to populations with different demographic profiles or WaSH conditions. These limitations should be considered alongside the model's strong predictive capabilities when evaluating its potential application in public health practice. Future research could include validating the model using different datasets from varying geographical contexts to further assess generalizability and potential overfitting issues. |

**Reviewer #1 Comments:**

| **Comment** | **Response** |
| --- | --- |
| 1. To be more informative, in Table 5, please show the stratification of the independent variables based on the stunting state. | Thank you for this suggestion. We have modified this table to show descriptive characteristics of the dataset stratified by stunting status (stunted vs. not stunted as two additional columns). This provides a clearer picture of how the independent variables differ between children who are stunted and those who are not. |
| 2. Authors are recommended to add some discussion on the impact of non-WaSH demographic variables on stunting based on their machine learning modeling. | We have added the following paragraph to the Discussion section, to discuss our results related to non-WaSH demographic variables:  Our results also reinforce the importance of several demographic variables for child growth, which were retained in the XGBoost model: female caregiver education, household caste/tribe, livestock ownership, household wealth, child’s gender, and child’s age. Globally, extensive evidence exists for the role of female caregiver education and household wealth as basic or enabling determinants of child nutritional status [Smith et al., 2015; Vaivada et al., 2020; Victora et al., 2021]. Similarly, household caste/tribe status and livestock ownership may be closely linked to household wealth [Zacharias et al., 2011; Khan et al., 2021]. The child’s gender may be important due to gendered social norms related to child feeding, particularly in India [Jayachandran et al., 2011; Mishra et al., 2004]. Finally, child age is known to predict linear growth, as most linear growth faltering occurs in the first 1000 days of life [Victora et al., 2021]. While these results align well with existing evidence, it was surprising that the XGBoost model recommended dropping the variable for optimal child feeding, given the clear importance of child feeding practices for growth. This exemplifies a key distinction between predictive modeling and causal inference frameworks. In our predictive approach, XGBoost prioritized variables that maximize predictive accuracy rather than establishing causal importance. The complex non-linear relationships and interactions captured by XGBoost may have determined that other variables in the model collectively provided stronger predictive signals for stunting outcomes in this particular dataset, even while child feeding remains theoretically important. |

**Reviewer #2 Comments:**

| **Comment** | **Response** |
| --- | --- |
| 1. The advances beyond [25] are unclear. As the authors state several times, the key factors influencing stunting remain the same, and the same algorithm appears to be the most optimal. Please clarify the advances. | Thank you for this important point. We have added a paragraph in the “Introduction” section that articulates the advances beyond our previous work (see below):  This study applies machine learning techniques to predict childhood stunting based on WaSH behaviors and infrastructure, addressing limitations of our previous structural equation modeling (SEM) approach. While the previous approach effectively identified pathways between WaSH factors and height-for-age z-scores, it has specific constraints: it primarily models linear relationships, requires strong theoretical assumptions about causal structures. In contrast, our machine learning approach offers certain advantages: (1) algorithms like XGBoost can capture complex non-linear relationships and interactions between WaSH variables without requiring pre-specified structural assumptions; (2) it quantifies predictive performance with metrics directly relevant to field applications such as sensitivity (recall) and specificity; (3) it identifies the combination of WaSH factors that maximizes prediction accuracy rather than focusing on individual pathways; and (4) it provides a framework for developing practical screening tools that could identify high-risk children in resource-limited settings. By systematically comparing multiple algorithms and feature engineering techniques, this study transforms insights about WaSH-stunting relationships into actionable tools for targeted intervention. |
| 2. While many different algorithms were used to predict stunting and the results from each reported, there is no explanation or discussion on why they think XGBoost performed better than others or why forward selection was the optimal feature engineering technique. | We have added a detailed explanation of why XGBoost performed better than other algorithms in the “Discussion” section of the revised manuscript as follows:  XGBoost's superior performance across all feature engineering approaches can be attributed to several factors. First, XGBoost builds sequential decision trees that learn from the errors of previous trees (gradient boosting), making it particularly effective at capturing non-linear relationships and interactions between WaSH variables. Second, XGBoost has built-in regularization parameters that help prevent overfitting, which is especially important in our relatively small dataset. Third, XGBoost handles mixed data types efficiently, accommodating our combination of binary, categorical, and continuous WaSH variables.  We have also added an explanation of why forward selection was particularly effective as follows:  Regarding forward selection's effectiveness, this technique likely performed well because it prioritizes features with the strongest predictive power while systematically excluding redundant or uninformative variables. Forward selection's iterative approach aligns well with the gradient boosting process of XGBoost, potentially creating synergy between the feature selection method and the algorithm. Furthermore, forward selection identified a parsimonious set of features that reduced noise in the data, allowing the XGBoost algorithm to focus on the most informative signals for stunting prediction. |
| 3. There is also no commentary on what are the difficulties in linking WaSH interventions with improvements in child growth. Why has this link been hard to determine in the past? | Thank you for raising this important point. We have added the following text in the Discussion section to explain why WaSH interventions have had difficulty with improving child growth in the past:  Researchers have hypothesized that this lack of effect on child growth may be because the WaSH interventions provided in those trials consisted of basic, low-cost infrastructure such as pit latrines, which may be insufficiently effective for reducing fecal contamination in the environment [Cumming et al., 2019; Pickering et al., 2019]. Partly in response to those trials, recommendations now focus on comprehensive, ‘transformative’ WaSH approaches, including high community-level coverage of improved sanitation combined with continuous and convenient access to drinking water [Pickering et al., 2019]. |
| 4. A primary concern with linear models is that if correlations exist between the covariates, feature importance and predictions can be unstable. I wonder if the covariates used in this analysis are correlated, and if so, these should be accounted for. One can use orthogonalization techniques like QR decomposition to disassociate the variables. | Thank you for this thoughtful comment about potential correlations between covariates. While QR decomposition is indeed a valuable technique for orthogonalizing variables, we chose to use Variance Inflation Factor (VIF) analysis, which is widely recognized as the standard diagnostic method for multicollinearity in regression analysis [Belsley et al., 2005; O'Brien, 2007]. VIF directly quantifies how much the variance of a regression coefficient is inflated due to multicollinearity with other predictors, making it particularly well-suited for our study's objectives.  We conducted a thorough VIF analysis for all features in our dataset, and have added a description of this analysis in the Methods section of the revised manuscript as follows:  The results confirmed that multicollinearity was not a major concern, as no variable exceeded the commonly used threshold of VIF > 5 [O'Brien, 2007]. The highest VIF values were observed for sanitation-related variables (improved sanitation: 4.25 and proportion of household members using improved sanitation: 3.81), which show moderate correlation but remain below problematic levels.  Additionally, we chose to maintain the original feature space rather than applying orthogonalization techniques like QR decomposition because: (1) the interpretability of our original features was critical for deriving actionable public health insights; (2) our machine learning approach, particularly XGBoost, is inherently robust to moderate multicollinearity [Chen & Guestrin, 2016]; and (3) our regularization techniques already provide effective mitigation for the levels of correlation present in our data. |
| 5. It seems that forward selection also found some demographic covariates to be important—these should also be discussed. | We have added the following paragraph to the Discussion section, to interpret and explain our results related to demographic covariates:  Our results also reinforce the importance of several demographic variables for child growth, which were retained in the XGBoost model: female caregiver education, household caste/tribe, livestock ownership, household wealth, child’s gender, and child’s age. Globally, extensive evidence exists for the role of female caregiver education and household wealth as basic or enabling determinants of child nutritional status [Smith et al., 2015; Vaivada et al., 2020; Victora et al., 2021]. Similarly, household caste/tribe status and livestock ownership may be closely linked to household wealth [Zacharias et al., 2011; Khan et al., 2021]. The child’s gender may be important due to gendered social norms related to child feeding, particularly in India [Jayachandran et al., 2011; Mishra et al., 2004]. Finally, child age is known to predict linear growth, as most linear growth faltering occurs in the first 1000 days of life [Victora et al., 2021]. While these results align well with existing evidence, it was surprising that the XGBoost model recommended dropping the variable for optimal child feeding, given the clear importance of child feeding practices for growth. This exemplifies a key distinction between predictive modeling and causal inference frameworks. In our predictive approach, XGBoost prioritized variables that maximize predictive accuracy rather than establishing causal importance. The complex non-linear relationships and interactions captured by XGBoost may have determined that other variables in the model collectively provided stronger predictive signals for stunting outcomes in this particular dataset, even while child feeding remains theoretically important. |
| 6. SEM has never been explained, and references for all the algorithms used are missing. | Thank you for noting this omission. We have added an explanation of SEM in the Methods section as follows:  SEM is a multivariate statistical framework that combines factor analysis and multiple regression to analyze structural relationships between measured variables and latent constructs [Kline, 2015]. SEM allows for the simultaneous estimation of multiple and interrelated dependencies, accounting for measurement error and enabling the modeling of complex causal pathways [Bollen, 1989].  We have also added references for all algorithms (See Table 3), including: logistic regression [Hosmer et al., 2013], classification tree [Breiman et al., 1984], SVM [Cortes & Vapnik, 1995], neural network [Haykin, 1998], and XGBoost [Chen & Guestrin, 2016]. |
| 7. Despite advocating for SEM in terms of interpretability of the features, the authors do not provide any interpretations of its results. | We have added below interpretation of the SEM results from our previous study in the “Discussion” section of the revised manuscript:  Our previous SEM analysis identified significant pathways from improved sanitation coverage, piped water coverage, and drinking water availability to height-for-age z-scores, mediated through household sanitation use. The SEM approach revealed that increased use of improved sanitation facilities was the most proximal determinant of improved HAZ, with village-level sanitation coverage having both direct effects and indirect effects (through household use) on child growth outcomes.  We have also added below text explaining how these SEM insights complement our machine learning findings:  In the current study, we used these SEM insights to inform feature selection, leveraging the identified mechanistic pathways. The interpretative value of the SEM approach lies in its ability to distinguish between direct and indirect effects and to model the sequential nature of WaSH pathways affecting child growth. For example, the SEM results suggested that handwashing stations may influence child growth through indirect pathways related to reduced pathogen transmission, rather than having a strong direct effect. These insights complement the predictive power of our machine learning models by providing potential explanations for why certain WaSH factors emerge as important predictors. |
| 8. Please provide a reference for SMOTE and a brief description. It seems to be very crucial for model training. | We have added a reference for SMOTE [Chawla et al., 2002] and expanded the description of this technique in the Methods section as follows:  SMOTE works by creating synthetic examples of the minority class (stunted children) in feature space rather than simply duplicating existing examples. The algorithm selects a minority class instance and its *k* nearest neighbors, then generates new synthetic instances along the line segments joining the selected instance to its neighbors. This approach provides the learning algorithm with a more balanced distribution of classes, potentially improving the model's ability to correctly classify minority instances. We applied SMOTE only to the training data in each fold of cross-validation, ensuring that the test data remained unmodified to provide an unbiased evaluation of model performance. |
| 9. How would the results change if no feature engineering was performed? | We have added a paragraph in the “Model Performance Results” section discussing this question as follows:  The impact of using no feature engineering versus various feature selection techniques can be observed in Table 7. When no feature engineering was performed (labeled as "None" in Table 7), the XGBoost model achieved an AUROC of 0.848, which was substantially lower than its performance with forward selection (AUROC 0.959). This improvement demonstrates the significant value of appropriate feature selection for complex algorithms like XGBoost. In contrast, simpler models showed minimal sensitivity to feature engineering. For instance, logistic regression maintained nearly identical performance with no feature engineering (AUROC 0.678) compared to any feature selection technique (AUROC range: 0.681-0.688). Classification trees performed slightly better with no feature engineering (AUROC 0.563) than with SEM-based feature selection (AUROC 0.558). SVM and neural networks showed modest improvements with feature selection compared to using all features, but these gains were much less dramatic than those observed with XGBoost. These comparative results highlight that while feature engineering is critical for optimizing complex non-linear models like XGBoost, it provides limited benefits for simpler, more rigid algorithms in this application. |
| 10. How was lambda determined in L2 regularization? | In our logistic regression model, we used L2 regularization (Ridge) with the default regularization strength parameter (C=1.0, which corresponds to λ=1.0, since C=1/λ). No specific cross-validation was performed to select this value; the default scikit-learn parameter was used.  We added verbiage in Table 3 under “Implementation Details” column for “Logistic Regression” row to indicate lambda value used in L2 regularization. |
| 11. Lines 287-289: Since [25] shows similar results using ML techniques, I am not sure this claim is true. | Thank you for this observation. We have removed this line in the revised manuscript. |
| 12. Lines 131-132: "Availability of the preferred drinking water source was a binary variable defined as having experienced source unavailability for at least 24 hours in the previous 2 weeks, or at any time in the previous 24 hours." Are these two conditions different? | We have revised this sentence to clarify that this binary variable was created from two questions, which aimed to capture different levels of severity of water unavailability (being unavailable for any length of time or for more than one full day). The sentence now reads as follows:  Availability of the preferred drinking water source was a binary variable created from two questions that asked whether the household had experienced source unavailability for at least one full day in the previous 2 weeks, or for any amount of time in the previous 24 hours. |
| 13. Line 149: "Standardized household wealth index, calculated using principal components analysis as described previously." How was this performed? A brief description would improve clarity. | We have added a clearer description of the household wealth index calculation (see below):  Standardized household wealth index was calculated using principal components analysis (PCA) of household assets and characteristics [Reese et al, 2019]. In this previous study, PCA was performed on variables including household asset ownership (chair, table, refrigerator, mattress, pressure cooker, scooter or motorcycle, mobile phone, electric fan, sewing machine and television), housing characteristics, agricultural land acreage owned and below poverty-line status [Reese et al, 2019]. |
| 14. Table 5 could be moved to the appendix, as its contribution to the main results are minor. | Thank you for the suggestion. We believe it’s helpful to retain this table in the main text because:   1. It provides essential descriptive statistics that establish baseline characteristics of our study population. Having this information in the main text allows readers to easily interpret subsequent analyses without navigating to the appendix. 2. Following Reviewer #1's suggestion, we've modified this table with stratification by stunting status. |

Thank you again for your thoughtful comments and suggestions, which helped us improve the quality and clarity of our manuscript. We hope that the revised version addresses the concerns raised and meets the standards for publication in PLOS ONE.

Sincerely,

Dr. Sheela Sinharoy

Hubert Department of Global Health, Rollins School of Public Health, Emory University

Email: [sssinh2@emory.edu](mailto:sssinh2@emory.edu)

**Amended Funding Statement**

This work was supported by the Bill & Melinda Gates Foundation [grant numbers OPP1008048 and OOP1125067]. The funder had no role in the study design, data collection, analysis, preparation of the manuscript, or decision to publish. There was no additional external funding received for this study.

**Amended Acknowledgements**

We thank the study team and participants in the original matched cohort study, without whom this work would not be possible. We also appreciate Shubhayu Sinharoy for his advice and insights during the project.
