# Supplementary material for: Applying machine learning to predict stunting in children under 5 years old based on water, sanitation and hygiene behaviors and infrastructure
Source: PLoS One. 2026 Mar 5;21(3):e0343796. doi: 10.1371/journal.pone.0343796 (PMC12962480; doi:10.1371/journal.pone.0343796)
Supplement: Attachment — Submitted filename: Response to Reviewers_2.docx [file pone.0343796.s005.docx]

**Response to Reviewers**

Dear Dr. Ashish Wasudeo Khobragade,

Thank you for your feedback on our manuscript titled "Applying machine learning to predict stunting in children under 5 years old based on water, sanitation and hygiene behaviors and infrastructure" (PONE-D-24-38002R1). We appreciate the opportunity to revise our work according to your suggestions and those of the reviewers. We have carefully addressed all comments and concerns in this revision. Below, we provide point-by-point responses to each comment.

**Journal Requirements:**

| **Comment** | **Response** |
| --- | --- |
| If the reviewer comments include a recommendation to cite specific previously published works, please review and evaluate these publications to determine whether they are relevant and should be cited. There is no requirement to cite these works unless the editor has indicated otherwise. | Not applicable |
| Please review your reference list to ensure that it is complete and correct. If you have cited papers that have been retracted, please include the rationale for doing so in the manuscript text, or remove these references and replace them with relevant current references. Any changes to the reference list should be mentioned in the rebuttal letter that accompanies your revised manuscript. If you need to cite a retracted article, indicate the article’s retracted status in the References list and also include a citation and full reference for the retraction notice. | We confirm that we reviewed the reference list to ensure that it is complete and correct. |

**Reviewer #3 Comments:**

| **Comment** | **Response** |
| --- | --- |
| The study employed five machine learning algorithms (including XGBoost, logistic regression, SVM, neural networks, classification trees). It uses four feature selection methods (SEM, forward selection, backward elimination, LASSO). | We acknowledge Reviewer #3’s general assessment, and note the absence of specific major/minor comments from this reviewer. |

**Reviewer #4 Major Comments:**

| **Major Comment** | **Response** |
| --- | --- |
| Clarity on Generalizability and External Validation: The study uses data from a single region (Odisha). The authors acknowledge limitations in generalizability but do not attempt external validation or even internal validation via holdout sets beyond cross-validation. Please clarify whether an independent holdout set was used. If not, discuss how model performance might be overestimated due to lack of external validation. Additionally, the manuscript should discuss more explicitly how these findings might or might not extrapolate to other contexts (e.g., sub-Saharan Africa, Southeast Asia). | We thank the reviewer for this important methodological question. To clarify, we did not use an independent holdout set beyond our 10-fold cross-validation approach. We appreciate the opportunity to discuss the potential implications of this choice and why we believe our validation approach remains robust for our study objectives.  Regarding potential overestimation of model performance, we acknowledge this is a valid theoretical concern. Cross-validation without an independent holdout set can lead to some optimistic bias because the entire dataset contributes to model selection decisions. However, several factors support the validity of our approach:  First, our rigorous cross-validation protocol - applying SMOTE only to training folds while keeping test folds unmodified - follows established best practices that help minimize potential bias.  Second, our primary finding (XGBoost's superiority over other algorithms) relies on relative performance comparisons under identical validation conditions rather than absolute performance metrics. Any potential overestimation would affect all algorithms similarly, preserving the validity of our comparative conclusions.  Third, for our dataset size (n=1,196), creating additional holdout sets would substantially reduce training data, potentially compromising model development. This represents an important trade-off in public health research where sample sizes are often constrained.  Regarding generalizability to other contexts, we have expanded our limitations section to more explicitly address how these findings might extrapolate to different settings. We have added the following text to our limitations:  "The geographic specificity of our study (rural Odisha, India) limits the direct extrapolation of our findings to other contexts. While the machine learning methodology demonstrated here is broadly applicable, the specific predictive relationships between WaSH factors and stunting may vary across contexts. The determinants of stunting are complex, multi-level, and interconnected [53] and, therefore, may exhibit different predictor importance rankings by location. Future research should validate these machine learning approaches using locally relevant datasets to ensure culturally and contextually appropriate risk prediction models."  We have also added the following statement regarding potential performance overestimation: "Our cross-validation approach, while following established best practices, may result in modest overestimation of model performance compared to external validation. However, this potential limitation does not undermine our primary contribution of demonstrating the comparative effectiveness of different machine learning algorithms under identical validation conditions." |
| Treatment of Missing Data:  The authors excluded a large proportion of children (630/1826, ~35%) due to missing predictor variables. While this is described, the implications for bias should be addressed more directly. Were there systematic differences between included and excluded children? A comparison table of included vs. excluded observations would strengthen the manuscript. | We appreciate the reviewer's important concern about potential selection bias from excluding 630 children (34.5% of those with anthropometric measurements) due to missing predictor variables. We acknowledge this represents a methodological consideration that warrants discussion.  We selected complete-case analysis over imputation based on several methodological advantages specific to machine learning applications. Tree-based algorithms like XGBoost and classification trees perform optimally with observed data patterns, as imputed values can introduce artificial relationships that may reduce model accuracy and real-world applicability. Our approach enables the development of a robust prediction tool based on genuine observed patterns that can be confidently applied in field settings where complete information is systematically collected. This methodology aligns with established practices in similar machine learning studies for child nutrition, including recent work by Anku & Duah (2024), Shen et al. (2023), and Bitew et al. (2022), supporting the validity of our approach.  Our analytic strategy focuses the model's applicability to scenarios where comprehensive WaSH and demographic data are available - a common situation in well-designed programmatic settings and intervention planning contexts. This represents a practical strength for implementation purposes, as the model can provide reliable predictions in settings where systematic data collection protocols are in place. The approach ensures that prediction accuracy is maximized for the target implementation context.  Our complete-case approach provides conservative and interpretable results with a substantial sample size of 1,196 children, which provides adequate statistical power for reliable model development and evaluation. The cross-validation methodology and comprehensive performance metrics demonstrate robust model performance and validate the effectiveness of our analytic approach. The focus on complete observations ensures that our predictive relationships reflect genuine patterns observable in practice, providing a solid foundation for the machine learning models presented in this study.  To address the reviewer's concern, we propose the following additions to the manuscript:  Methods Section: "Our analytic dataset consisted of 1,196 children with complete information across all predictor variables, representing 65.5% of children with anthropometric measurements. This complete-case analysis approach was selected to optimize machine learning model performance, as tree-based algorithms like XGBoost and classification trees perform best with observed rather than imputed data patterns, ensuring reliable prediction accuracy for practical applications in programmatic settings."  Discussion Section: " Another limitation is our use of the complete-case analysis approach. The complete-case analysis approach, while methodologically appropriate for machine learning applications, means our findings are most directly applicable to populations where systematic WaSH and demographic data collection is feasible. While we cannot rule out potential selection bias from excluding observations with missing data, this represents a common scenario in well-designed program implementation contexts. Future validation studies across diverse populations and data collection contexts would further establish the broader applicability of our predictive modeling approach." |
| Feature Importance and Interpretability:  Although XGBoost achieved high predictive accuracy, its interpretability is more limited than simpler models. The manuscript should present feature importance rankings or SHAP values to clarify which predictors contributed most to model predictions. This is especially relevant since the Discussion emphasizes the practical value of identifying key WaSH variables. Visuals (e.g., variable importance plots) would improve readability. | We appreciate the reviewer's suggestion regarding feature importance and interpretability. Our current approach already achieves the key interpretability objectives that SHAP analysis would provide, while being more appropriate for our study's public health applications.  Our comprehensive feature engineering approach, which systematically compared four different variable selection methods (SEM, forward selection, backward elimination, and LASSO), serves as a robust method for assessing feature importance across multiple analytical frameworks. Each method provides insights into variable importance from different perspectives: forward selection identifies variables with strongest individual predictive power, backward elimination reveals variables whose removal most degrades performance, LASSO provides regularization-based importance through coefficient shrinkage, and SEM offers theory-driven importance based on established causal pathways. The convergence of results across these methods—particularly the consistent identification of improved sanitation coverage, handwashing stations, piped water coverage, and water source availability as key predictors, provides stronger evidence of true feature importance than any single explanation method could offer. This multi-method validation approach inherently provides the global interpretability insights that SHAP analysis would deliver, while offering additional confidence through methodological triangulation.  For public health applications focused on intervention targeting - which is our primary objective - global feature importance is more relevant than individual prediction explanations that SHAP provides. Public health practitioners need to understand which WaSH factors are most predictive across populations to design targeted interventions, not explanations for individual predictions. Our identification of the four most predictive WaSH variables directly serves this need.  Furthermore, SHAP analysis is most valuable when the goal is explaining individual predictions or understanding complex feature interactions. Our study's objectives center on comparative algorithm performance and identifying key predictive variables for intervention design. The multi-method feature engineering process has already accomplished this by systematically identifying which variables contribute most to predictive accuracy when considered together across different analytical approaches. |
| Choice of Performance Metrics: The primary metric reported is AUROC, which is appropriate but can be misleading in imbalanced datasets. Consider providing precision-recall curves and reporting the area under the precision-recall curve (AUPRC) for a fuller understanding of the model’s utility. Also, please clarify whether model thresholds were optimized for specific sensitivity/specificity tradeoffs. | We thank the reviewer for this thoughtful suggestion regarding precision-recall curves and additional performance metrics. While we recognize the value of these approaches, we believe our current comprehensive evaluation provides sufficient insight for our study objectives and comparative analysis.  AUROC remains the gold standard for binary classification evaluation and is particularly valuable for comparing across different algorithms, which was a primary objective of our study. While AUROC can be influenced by class imbalance, we addressed this through SMOTE resampling, making AUROC a valid primary metric for our comparative analysis.  Our comprehensive evaluation already includes precision, recall (sensitivity), specificity, accuracy, and F1-score (Table 7), providing a complete picture of model performance across multiple dimensions. These metrics collectively address the reviewer's concerns about model utility in imbalanced datasets. The F1-score, in particular, provides a balanced measure that accounts for both precision and recall, making it well-suited for evaluating performance in the presence of any remnant class imbalance.  Adding precision-recall curves and AUPRC would not fundamentally change our conclusions about XGBoost's superior performance, as evidenced by its consistent superiority across all reported metrics. Our study's primary contribution lies in demonstrating the comparative effectiveness of different machine learning algorithms and feature engineering approaches for stunting prediction, rather than optimizing a single model for deployment.  The threshold-independent nature of AUROC makes it particularly appropriate for our comparative study design, as it evaluates model performance across all possible classification thresholds rather than at a single operating point. This approach aligns with our objective of identifying the most promising algorithm-feature engineering combinations for future development and deployment in specific public health contexts. |
| Ethical and Data Sharing Considerations:  The data availability statement indicates that data can be obtained from the corresponding author on reasonable request. PLOS ONE requires data to be publicly available unless there are legal or ethical restrictions. Please clarify whether the data repository can be shared openly (e.g., via Dryad or other repositories) or whether formal approval is required to access the data. | Please see our prior response to the Editor dated 6 April 2025 which is reproduced once again below:  The data underlying the findings described in this manuscript have been uploaded to FigShare. They can be accessed via the following private link, which will be updated to a publicly available DOI upon acceptance: <https://figshare.com/s/5678f9122fcc78739a2d> |

**Reviewer #4 Minor Comments:**

| **Minor Comment** | **Response** |
| --- | --- |
| Terminology Consistency: Throughout the manuscript, the authors sometimes refer to the outcome as "stunting" and elsewhere as "HAZ" (height-for-age z score). For clarity, consistently refer to the binary outcome (stunted/not stunted) in the context of prediction. | We appreciate the reviewer's attention to terminology consistency. However, we believe there may be a misunderstanding about our use of these terms that we would like to clarify:  Throughout our current manuscript, we consistently refer to our outcome variable as "stunting" - the binary classification (stunted/not stunted) that serves as our prediction target. We define this clearly in our Methods section: "According to the 2006 World Health Organization (WHO) criteria, a child is considered stunted if their LAZ/HAZ score is more than 2 standard deviations below the WHO growth standard. In our analysis, stunting is represented as a binary variable: children identified as stunted are assigned a code of 1, while others are coded as 0."  The references to "height-for-age z-scores" (HAZ) in our manuscript occur specifically when discussing our previous structural equation modeling (SEM) study [Reese et al., 2019], which used the continuous HAZ measure as its outcome variable. For example, we state: "It is worth noting that the previous study used HAZ as the outcome variable rather than stunting." This distinction is scientifically important and intentional, as it accurately represents the methodological differences between our current machine learning approach (predicting binary stunting status) and our previous SEM approach (modeling continuous height-for-age z-scores).  When we mention HAZ in the context of calculating stunting (e.g., "Stunting was calculated based on length/height-for-age z score"), this is appropriate because we are explaining the measurement process - how the continuous z-score is transformed into the binary stunting classification that serves as our prediction outcome.  We believe this terminology use is both consistent and scientifically accurate, clearly distinguishing between our current binary outcome (stunting) and the continuous measure used in our previous work (HAZ), while appropriately explaining the relationship between these measures. |
| Tables and Figures: Tables 6 and 7 are quite dense. Consider simplifying or moving details to supplementary material. A flowchart summarizing sample inclusion/exclusion would be helpful. | We appreciate the reviewer's suggestions regarding table presentation and sample flow visualization. We have created a flowchart (Appendix 1) that clearly illustrates the progression from the original study population to our final analytic dataset, showing the exclusion of 630 children due to missing data and the retention of 1,196 children for analysis.  Regarding Tables 6 and 7, we believe these should remain in the main manuscript for several important reasons. Table 6 presents the core methodological foundation of our feature engineering approach, showing how four different variable selection techniques (SEM, forward selection, backward elimination, and LASSO) identified different subsets of predictors. This information is essential for readers to understand why certain variables were retained or excluded in our final models.  Similarly, Table 7 contains our primary findings—the comparative performance of all algorithm-feature engineering combinations across six key metrics (AUROC, recall, specificity, accuracy, precision, and F1-score). This table directly supports our main conclusions about XGBoost's superior performance and the effectiveness of forward selection.  We have ensured that both tables are formatted clearly and contain only information directly relevant to our primary research questions. |
| Literature Context: The Discussion references prior trials showing mixed effects of WaSH interventions. Including brief reflections on why machine learning predictions may differ from intervention effects (prediction vs. causation) would strengthen the argument. | In line with the reviewer’s suggestion, we propose adding the following sentence to the paragraph in the Discussion where we discuss other WaSH interventions:  “Still, our results are focused on predictive modeling, which may differ from intervention effects under real-world conditions; gold standard evidence from randomized controlled trials of ‘transformative’ WaSH approaches would further strengthen the evidence base related to causal linkages between WaSH and child stunting.” |
| Typographical Issues: Some minor typographical errors (e.g., inconsistent spacing, capitalization in section headings) should be corrected before publication. | Thank you for pointing this out. We have now corrected the typographical errors in the manuscript. |

Additionally, please refer to your email note dated 22 July 2025 in which you advised us to ignore the comments from **Reviewer #5** as these were irrelevant. The basis of the irrelevance of Reviewer #5’s comments are provided in the appendix of this rebuttal latter.

Thank you again for your thoughtful comments and suggestions, which helped us improve the quality and clarity of our manuscript. We hope that the revised version addresses the concerns raised and meets the standards for publication in PLOS ONE.

Sincerely,

Dr. Sheela Sinharoy

Hubert Department of Global Health, Rollins School of Public Health, Emory University

Email: [sssinh2@emory.edu](mailto:sssinh2@emory.edu)

**Appendix**

Email from PLOS ONE dated 22 July 2025:

From: **plosone** <[plosone@plos.org](mailto:plosone@plos.org)>
Date: Tue, Jul 22, 2025 at 11:41 AM
Subject: Re: PLOS ONE Decision: Revision required [PONE-D-24-38002R1]
To: [sanayasm97@gmail.com](mailto:sanayasm97@gmail.com) <[sanayasm97@gmail.com](mailto:sanayasm97@gmail.com)>

Dear Dr. Sinharoy, 

I hope this email finds you well.

Please see below email from Academic Editor in response to your query regarding reviewer comments.

***

Thank you for your email.
Dear Authors, please ignore the irrelevant comments made by Reviewer 5. 
Thanks and regards

***

Thank you and if you need further assistance, please let me know. 

Kind regards,

Johnelle Ryan Razo
Straive Editorial Assistant

PLOS ONE | [plosone@plos.org](mailto:plosone@plos.org)

Empowering researchers to transform science

Case Number: 09125251

Email to the Editor dated 16 July 2025:

**From:** Sanaya Sinharoy [[sanayasm97@gmail.com](mailto:sanayasm97@gmail.com)]
**Sent:** 7/16/2025, 4:36 AM
**To:** [plosone@plos.org](mailto:plosone@plos.org)
**Subject:** Re: PLOS ONE Decision: Revision required [PONE-D-24-38002R1] - [EMID:5af431c330e6b6ce]

Dear Dr. Khobragade and PLOS ONE Editorial Team,

Thank you for sending the reviewer comments for our manuscript "Applying machine learning to predict stunting in children under 5 years old based on water, sanitation and hygiene behaviors and infrastructure" (PONE-D-24-38002R1).

We have carefully reviewed all three sets of reviewer comments and would like to respectfully bring to your attention what appears to be a significant mix-up with Reviewer #5's comments.

**Evidence of the Mix-up:**

The comments from Reviewer #5 appear to be intended for a completely different manuscript, as evidenced by several specific inconsistencies:

1. **Title Discussion**: The reviewer states: *"This title is brief and communicates the key elements of the study, which focuses on 'brain structure and function' and 'emotional responsiveness and depression risk.' It would be better, though; if there were inclusions of additional methodology or population sample description (e.g., fMRI, longitudinal, adolescents)."* Our manuscript title contains no reference to brain structure, emotional responsiveness, depression, fMRI, or adolescents - it focuses on machine learning prediction of stunting in children based on WaSH factors.
2. **Abstract Comments**: The reviewer mentions the abstract being "dense with jargon" related to neuroimaging and affective neuroscience, which are completely unrelated to our public health/nutrition study.
3. **Methodology Comments**: References to "neuroimaging," "imaging protocols," "behavioral tasks," "fMRI measures," and "LMMs" (presumably linear mixed models in a neuroimaging context) are not relevant to our machine learning analysis of WaSH and stunting data.
4. **Results Comments**: Discussion of "neural conclusions," "behavioral and neural conclusions," and "neural patterns of activation" are entirely unrelated to our findings about XGBoost performance and WaSH predictors.
5. **General Content**: Throughout the review, there are references to depression risk, emotional processing, affective neuroscience, and brain imaging - none of which are topics covered in our manuscript.

**Request:**

Could you please verify and send us the correct comments from Reviewer #5 for our manuscript? We want to ensure we address all relevant feedback appropriately in our revision.

The comments from Reviewers #3 and #4 appear to be correctly matched to our manuscript and we will address those accordingly.

Thank you for your time and assistance in resolving this matter.

Best regards,

Sanaya Sinharoy
