# Supplementary material for: Applying machine learning to predict stunting in children under 5 years old based on water, sanitation and hygiene behaviors and infrastructure
Source: PLoS One. 2026 Mar 5;21(3):e0343796. doi: 10.1371/journal.pone.0343796 (PMC12962480; doi:10.1371/journal.pone.0343796)
Supplement: Attachment — Submitted filename: General Comments.docx [file pone.0343796.s004.docx]

**Abstract**

- **Clarity of Aim**: The objective is well stated, but the abstract should more explicitly link machine learning predictions to potential public health action, beyond “informing targeted interventions.”
- **Methods Description**: While feature engineering and algorithms are listed, the rationale for choosing them is not explained. A simpler description may improve accessibility for non-technical readers.
- **Results Presentation**: Reporting accuracy, sensitivity, specificity, and AUROC is good, but including the sample size (n = 1,196) in the abstract would strengthen context.
- **Conclusions**: The conclusion claims strong predictive power; however, the limitations (e.g., lack of external validation, exclusion of 35% of data due to missing variables) are not acknowledged here and should be briefly noted.

**Introduction**

- **Contextualization**: The introduction provides a strong rationale but could better differentiate between prediction vs. causation (machine learning predicts associations, not causal relationships).
- **Literature Gap**: While prior WaSH–stunting studies are cited, more emphasis on why machine learning is needed beyond SEM or regression models would strengthen the justification.
- **Overclaiming**: The introduction sometimes suggests that predictive modeling can directly inform interventions, but this could mislead readers into assuming causality.

**Methods**

- **Study Design & Dataset**: The decision to use only complete-case data (excluding ~35%) is justified, but the potential for bias should be discussed more thoroughly, possibly with a sensitivity analysis or comparison table.
- **Feature Selection**: Four different feature engineering methods are included, but the rationale for combining theory-driven SEM with purely data-driven methods is not fully justified.
- **Validation Strategy**: Reliance on 10-fold cross-validation without an independent holdout or external validation risks overestimating performance. This needs stronger discussion.
- **Missing Data Handling**: Instead of complete-case analysis, alternative imputation methods could have been tested and results compared. This limitation should be highlighted.
- **Ethics Statement**: Ethical approvals are reported appropriately, but since secondary data was used, clarification on household-level consent and anonymization should be more explicit.

**Results**

- **Descriptive Statistics**: The descriptive results are comprehensive, but some tables (e.g., Table 6, 7) are dense and could be simplified or moved to supplementary material.
- **Model Performance**: XGBoost outperformed other models, but overfitting risk should be acknowledged. Reporting only AUROC is limiting; precision-recall curves or AUPRC could provide additional insights, especially in imbalanced data.
- **Interpretability**: The identification of four WaSH predictors is useful, but feature importance measures (e.g., SHAP values or plots) would make findings more interpretable for practitioners.
- **Visualization**: Figures summarizing model comparison or feature importance are missing and would improve accessibility.

**Discussion**

- **Comparison to Prior Literature**: While the discussion refers to earlier SEM work, it could do more to explain why predictive results differ from intervention trial outcomes (prediction ≠ causal effect).
- **Generalisability**: The study acknowledges its single-region focus but could provide stronger discussion on cultural, infrastructural, and epidemiological differences across LMICs.
- **Bias and Limitations**: Selection bias due to missing data exclusion is acknowledged, but not fully explored. A comparison of included vs. excluded children (age, socio-economic status, WaSH access) would strengthen the validity.
- **Interpretability vs. Accuracy**: The discussion emphasizes XGBoost accuracy but should balance this with considerations of transparency and practical use in public health decision-making.

**Conclusion**

- **Overstatement**: The conclusion suggests WaSH factors “strongly predict” stunting, but this should be tempered with caution about generalizability and methodological limitations.
- **Policy Relevance**: A more explicit discussion of how these findings could be operationalized by policymakers or NGOs (e.g., risk screening tools, targeting high-risk households) would enhance applied value.

**Tables & Figures**

- **Complexity**: Some tables are too detailed for the main text; consider moving to supplementary files and replacing with summary visuals (e.g., model comparison plots, variable importance bar chart).
- **Flowchart**: A flowchart for sample inclusion/exclusion is mentioned but should be emphasized in the main text, not only in the appendix.

**Data Sharing & Transparency**

- **Data Availability**: The FigShare repository is appropriate, but a public DOI should be provided at acceptance. The current phrasing (“private link will be updated”) is not compliant with journal data policies.
