# Supplementary material for: Applying machine learning to predict stunting in children under 5 years old based on water, sanitation and hygiene behaviors and infrastructure
Source: PLoS One. 2026 Mar 5;21(3):e0343796. doi: 10.1371/journal.pone.0343796 (PMC12962480; doi:10.1371/journal.pone.0343796)
Supplement: Attachment — Submitted filename: Response_to_Reviewers_auresp_3.docx [file pone.0343796.s006.docx]

**Response to Reviewers**

Dear Dr. Ashish Wasudeo Khobragade,

Thank you for your feedback on our manuscript titled "Applying machine learning to predict stunting in children under 5 years old based on water, sanitation and hygiene behaviors and infrastructure" (PONE-D-24-38002R2). We appreciate the opportunity to revise our work according to your suggestions and those of the reviewers. We have carefully addressed all comments and concerns in this revision. Below, we provide point-by-point responses to each comment.

**Journal Requirements:**

| **Comment** | **Response** |
| --- | --- |
| If the reviewer comments include a recommendation to cite specific previously published works, please review and evaluate these publications to determine whether they are relevant and should be cited. There is no requirement to cite these works unless the editor has indicated otherwise. | Not applicable |
| Please review your reference list to ensure that it is complete and correct. If you have cited papers that have been retracted, please include the rationale for doing so in the manuscript text, or remove these references and replace them with relevant current references. Any changes to the reference list should be mentioned in the rebuttal letter that accompanies your revised manuscript. If you need to cite a retracted article, indicate the article’s retracted status in the References list and also include a citation and full reference for the retraction notice. | A new citation (Reference 54) was added, and the reference list numbering updated accordingly. This addition is mentioned in the rebuttal letter.  We confirm that we reviewed the reference list to ensure that it is complete and correct. |

**Reviewer #6 General Comments:**

| **General Comment** | **Response** |
| --- | --- |
| **Abstract**   - **Clarity of Aim**: The objective is well stated, but the abstract should more explicitly link machine learning predictions to potential public health action, beyond “informing targeted interventions.” - **Methods Description**: While feature engineering and algorithms are listed, the rationale for choosing them is not explained. A simpler description may improve accessibility for non-technical readers. - **Results Presentation**: Reporting accuracy, sensitivity, specificity, and AUROC is good, but including the sample size (n = 1,196) in the abstract would strengthen context. - **Conclusions**: The conclusion claims strong predictive power; however, the limitations (e.g., lack of external validation, exclusion of 35% of data due to missing variables) are not acknowledged here and should be briefly noted. | We thank the reviewer for the constructive feedback. We have addressed all four points as follows:  **Clarity of Aim:** We revised the objective to explicitly link predictions to public health action: "with the goal of identifying high-risk children who would benefit most from targeted interventions."  **Methods Description:** We simplified the rationale, noting that "Five machine learning algorithms commonly used for binary classification tasks were compared."  **Results Presentation:** We added the sample size in both Methods ("Complete data were available for 1,196 children") and Results ("Among 1,196 children analyzed...").  **Conclusions:** We added a statement acknowledging key limitations: "However, these findings require external validation in other populations, and the complete-case analysis approach (excluding 35% of children with missing data) may limit generalizability to settings with less systematic data collection. |
| **Introduction**   - **Contextualization**: The introduction provides a strong rationale but could better differentiate between prediction vs. causation (machine learning predicts associations, not causal relationships). - **Literature Gap**: While prior WaSH–stunting studies are cited, more emphasis on why machine learning is needed beyond SEM or regression models would strengthen the justification. - **Overclaiming**: The introduction sometimes suggests that predictive modeling can directly inform interventions, but this could mislead readers into assuming causality. | We thank the reviewer for these important observations about distinguishing prediction from causation and clarifying the role of machine learning.  **Contextualization:** We agree this distinction is critical. We have added explicit language in paragraph 3 stating: "However, it is important to note that machine learning models identify predictive associations rather than causal relationships; they can predict which children are at highest risk based on observed patterns, but do not establish that interventions targeting these predictors will necessarily improve outcomes."  **Literature Gap:** We have strengthened the justification for machine learning by adding clarification in paragraph 5 that distinguishes between the purposes of SEM (understanding causal mechanisms) versus ML (risk prediction for programmatic screening). We now explicitly state: "The SEM approach was well-suited for understanding causal pathways and mechanisms, whereas machine learning offers complementary advantages for predictive applications."  **Overclaiming:** We have revised language throughout to avoid implying causality. Key changes include: (a) replacing "inform targeted interventions" with "develop tools for identifying high-risk children" and "risk identification and targeted program planning"; (b) adding substantial text in paragraph 5 clarifying that "while our SEM analysis provided insights into causal mechanisms...the machine learning approach developed here focuses on prediction"; and (c) explicitly noting that "predictive models can inform programmatic decisions about resource allocation and screening priorities, but do not replace the need for rigorous causal inference methods when evaluating intervention effectiveness." |
| **Methods**   - **Study Design & Dataset**: The decision to use only complete-case data (excluding ~35%) is justified, but the potential for bias should be discussed more thoroughly, possibly with a sensitivity analysis or comparison table. - **Feature Selection**: Four different feature engineering methods are included, but the rationale for combining theory-driven SEM with purely data-driven methods is not fully justified. - **Validation Strategy**: Reliance on 10-fold cross-validation without an independent holdout or external validation risks overestimating performance. This needs stronger discussion. - **Missing Data Handling**: Instead of complete-case analysis, alternative imputation methods could have been tested and results compared. This limitation should be highlighted. - **Ethics Statement**: Ethical approvals are reported appropriately, but since secondary data was used, clarification on household-level consent and anonymization should be more explicit. | We thank the reviewer for these thoughtful suggestions to strengthen the Methods section.  **Study Design & Dataset:** We appreciate this important concern. While our original manuscript included a flow chart (Appendix 1) showing the exclusion of 35% of children and stated our rationale for complete-case analysis, we agree that potential bias warranted more thorough discussion. We have now added substantial text to the Data Source section explicitly acknowledging potential selection bias, and contextualizing how this limitation affects generalizability.  **Feature Selection:** We agree this needed clearer justification. We have added new text to the Feature Engineering section explaining our rationale: "We compared both theory-driven (SEM) and data-driven (forward selection, backward elimination, LASSO) approaches to feature selection for two reasons: (1) to assess whether theoretical knowledge from causal modeling (SEM) improves predictive performance compared to purely algorithmic selection, and (2) to determine which combination of feature engineering and machine learning algorithm yields optimal predictive accuracy for practical application."  **Validation Strategy:** We acknowledge this important limitation. While we followed established best practices using 10-fold cross-validation for algorithm comparison, we agree that the lack of external validation needed stronger discussion. We have added a new subsection titled "Model Validation Approach" that explicitly discusses this.  **Missing Data Handling:** While our original manuscript included rationale for complete-case analysis and discussed this limitation in the Discussion section, we agree it warranted more prominent treatment in Methods. We have now strengthened the discussion in the Data Source section (as noted in response to Comment 1) to more explicitly highlight how the complete-case approach may limit generalizability. The limitation of not testing imputation methods is now addressed both in the enhanced Methods text and in the existing Discussion section.  **Ethics Statement:** We have enhanced this section to be more explicit about consent and anonymization. We added clarifying language: "Informed written consent was obtained from the male and/or female heads of all enrolled households for participation in the original study, including collection of household survey data and child anthropometric measurements" and "All personal identifiers were removed from the dataset before transfer." |
| **Results**   - **Descriptive Statistics**: The descriptive results are comprehensive, but some tables (e.g., Table 6, 7) are dense and could be simplified or moved to supplementary material. - **Model Performance**: XGBoost outperformed other models, but overfitting risk should be acknowledged. Reporting only AUROC is limiting; precision-recall curves or AUPRC could provide additional insights, especially in imbalanced data. - **Interpretability**: The identification of four WaSH predictors is useful, but feature importance measures (e.g., SHAP values or plots) would make findings more interpretable for practitioners. - **Visualization**: Figures summarizing model comparison or feature importance are missing and would improve accessibility. | We thank the reviewer for these suggestions regarding presentation and interpretation of results.  **Descriptive Statistics:** We have retained Tables 6 and 7 in the main text as they present core findings essential to our study's contribution. Table 6 shows how different feature selection methods inform model development, which is essential for understanding our comparative approach. Table 7 presents the comprehensive performance comparison across all algorithm-feature engineering combinations, which is central to our study's core contribution. Given that readers may approach this paper specifically to compare algorithm performance for stunting prediction, these tables provide critical reference information that warrants main text placement.  **Model Performance:** We appreciate the concern about overfitting and have added text to the Model Performance Results section explicitly acknowledging this risk and describing our mitigation strategies (10-fold CV, hyperparameter tuning, built-in regularization).  Regarding precision-recall curves and AUPRC, we thank the reviewer for this thoughtful suggestion. While we recognize the value of these approaches, we believe our current comprehensive evaluation provides sufficient insight for our study objectives and comparative analysis. AUROC remains the gold standard for binary classification evaluation and is particularly valuable for comparing across different algorithms, which was a primary objective of our study. While AUROC can be influenced by class imbalance, we addressed this through SMOTE resampling, making AUROC a valid primary metric for our comparative analysis.  Our comprehensive evaluation already includes precision, recall (sensitivity), specificity, accuracy, and F1-score (Table 7), providing a complete picture of model performance across multiple dimensions. These metrics collectively address concerns about model utility in imbalanced datasets. The F1-score provides a balanced measure that accounts for both precision and recall, making it well-suited for evaluating performance in the presence of any remnant class imbalance.  Adding precision-recall curves and AUPRC would not fundamentally change our conclusions about XGBoost's superior performance, as evidenced by its consistent superiority across all reported metrics. Our study's primary contribution lies in demonstrating the comparative effectiveness of different machine learning algorithms and feature engineering approaches for stunting prediction, rather than optimizing a single model for deployment. The threshold-independent nature of AUROC makes it particularly appropriate for our comparative study design, as it evaluates model performance across all possible classification thresholds rather than at a single operating point. This approach aligns with our objective of identifying the most promising algorithm-feature engineering combinations for future development and deployment in specific public health contexts.  **Interpretability:** As detailed in our previous round of revisions, we believe our current approach already achieves the key interpretability objectives that SHAP analysis would provide, while being more appropriate for our study's public health applications.  Our comprehensive feature engineering approach, which systematically compared four different variable selection methods (SEM, forward selection, backward elimination, and LASSO), serves as a robust method for assessing feature importance across multiple analytical frameworks. Each method provides insights into variable importance from different perspectives: forward selection identifies variables with strongest individual predictive power, backward elimination reveals variables whose removal most degrades performance, LASSO provides regularization-based importance through coefficient shrinkage, and SEM offers theory-driven importance based on established causal pathways. The convergence of results across these methods- particularly the consistent identification of improved sanitation coverage, handwashing stations, piped water coverage, and water source availability as key predictors, provides stronger evidence of true feature importance than any single explanation method could offer. This multi-method validation approach inherently provides the global interpretability insights that SHAP analysis would deliver, while offering additional confidence through methodological triangulation.  For public health applications focused on intervention targeting - which is our primary objective - global feature importance is more relevant than individual prediction explanations that SHAP provides. Public health practitioners need to understand which WaSH factors are most predictive across populations to design targeted interventions, not explanations for individual predictions. Our identification of the four most predictive WaSH variables directly serves this need.  Furthermore, SHAP analysis is most valuable when the goal is explaining individual predictions or understanding complex feature interactions. Our study's objectives center on comparative algorithm performance and identifying key predictive variables for intervention design. The multi-method feature engineering process has already accomplished this by systematically identifying which variables contribute most to predictive accuracy when considered together across different analytical approaches.  **Visualization:** We appreciate this valuable suggestion. We have added a new figure (Figure 1) that visually compares AUROC performance across all five machine learning algorithms and four feature engineering approaches. The figure complements our comprehensive performance tables by providing an immediate visual summary of our comparative analysis, while the detailed tables retain all metrics for readers requiring complete information. |
| **Discussion**   - **Comparison to Prior Literature**: While the discussion refers to earlier SEM work, it could do more to explain why predictive results differ from intervention trial outcomes (prediction ≠ causal effect). - **Generalisability**: The study acknowledges its single-region focus but could provide stronger discussion on cultural, infrastructural, and epidemiological differences across LMICs. - **Bias and Limitations**: Selection bias due to missing data exclusion is acknowledged, but not fully explored. A comparison of included vs. excluded children (age, socio-economic status, WaSH access) would strengthen the validity. - **Interpretability vs. Accuracy**: The discussion emphasizes XGBoost accuracy but should balance this with considerations of transparency and practical use in public health decision-making. | We thank the reviewer for these thoughtful suggestions to strengthen the Discussion section.  **Comparison to Prior Literature (Prediction ≠ Causal Effect):** We agree this critical distinction warranted more explicit discussion. We have added substantial text to the paragraph discussing concordance with SEM results, explicitly explaining why strong predictive associations do not necessarily imply equivalent intervention effects. We illustrate this prediction-causation gap using examples from RCTs that found no stunting effects despite observational associations, and clearly articulate what our models CAN do (identify vulnerable children) versus what they CANNOT do (predict intervention effectiveness).  **Generalizability Across LMICs:** While a comprehensive discussion of cultural, infrastructural, and epidemiological differences across LMICs would be outside the scope of this work, we have added a couple of sentences to the Discussion to provide an illustrative example of these differences as they relate to our findings.  **Bias and Limitations (Selection Bias):** We have substantially enhanced our discussion of selection bias implications. In the Methods section, we revised text to acknowledge potential selection bias using careful conditional language: "If missing data patterns are systematic...several implications follow." In the Discussion, we expanded the limitations paragraph to explore how systematic missingness could affect model validity, including three specific implications: optimistic performance estimates, differential performance across populations, and varying predictive importance of WaSH factors depending on baseline conditions. We emphasize the need for external validation and performance monitoring across diverse subgroups.  **Interpretability vs. Accuracy:** We agree this trade-off deserved more explicit discussion. We have added a new paragraph that comprehensively addresses the balance between XGBoost's superior accuracy and its "black box" nature. The new text discusses how interpretability affects trust, stakeholder buy-in, and field implementation; contrasts XGBoost with simpler interpretable models like logistic regression; provides guidance on when each approach may be preferable based on implementation context; and suggests hybrid approaches for future implementations. This addition balances our emphasis on XGBoost's performance with practical considerations for public health decision-making. |
| **Conclusion**   - **Overstatement**: The conclusion suggests WaSH factors “strongly predict” stunting, but this should be tempered with caution about generalizability and methodological limitations. - **Policy Relevance**: A more explicit discussion of how these findings could be operationalized by policymakers or NGOs (e.g., risk screening tools, targeting high-risk households) would enhance applied value. | We thank the reviewer for these important suggestions to strengthen the Conclusion section.  **Overstatement:** We agree that more cautious language is appropriate given our study's limitations. We have revised the Conclusion to temper claims about predictive power and added explicit acknowledgment of key limitations. Specific changes include: (a) changing "can accurately predict" to "show promise for predicting" and adding geographic specificity "in rural Odisha, India"; (b) contextualizing performance with "In our dataset"; (c) changing "could significantly enhance" to "has potential to enhance"; and (d) adding an entire paragraph explicitly acknowledging limitations including the need for external validation, the single geographic context, the exclusion of 35% of children due to missing data, and the critical distinction that predictive models do not establish causal intervention effects.  **Policy Relevance:** We appreciate this valuable suggestion and have added substantial content on operationalization. We now include a new paragraph with concrete examples of how findings could be implemented in practice, including: (1) using simple data collection forms to capture the four key WaSH variables and generate risk scores, (2) prioritizing highest-risk households for intensive interventions when resources are limited, (3) targeting behavior change communication to families of high-risk children, and (4) allocating follow-up monitoring based on predicted risk levels. We explain how such risk-based targeting could improve program efficiency by focusing resources where they are most likely to impact child growth outcomes. We also note that future research should assess whether ML-informed targeting improves program outcomes in practice. |
| **Tables & Figures**   - **Complexity**: Some tables are too detailed for the main text; consider moving to supplementary files and replacing with summary visuals (e.g., model comparison plots, variable importance bar chart). - **Flowchart**: A flowchart for sample inclusion/exclusion is mentioned but should be emphasized in the main text, not only in the appendix. | We thank the reviewer for these suggestions regarding presentation.  **Complexity of Tables:** We appreciate this feedback and have carefully considered table placement. In response to the reviewer's earlier suggestion for visualization, we have added Figure 1, which provides a summary visual comparison of AUROC performance across all five machine learning algorithms and four feature engineering approaches. This figure makes the key comparative findings immediately accessible.  Regarding table placement, as noted in our response to Results comments, we have retained Tables 6 and 7 in the main text because they constitute our study's primary findings. Table 6 shows how different feature selection methods inform model development, which is essential for understanding our comparative approach and cannot be easily summarized in visual form. Table 7 presents comprehensive performance metrics (AUROC, recall, specificity, accuracy, precision, F1-score) across all 20 algorithm-feature engineering combinations. Given that readers may reference this paper specifically to compare algorithm performance for stunting prediction across multiple evaluation criteria, these detailed tables provide critical information that warrants main text placement. The addition of Figure 1 now provides the visual summary, while the tables retain complete information for readers requiring detailed metrics.  **Flowchart for Sample Inclusion/Exclusion:** We have revised the Methods section (Data Source subsection) to give more prominence to the sample selection process and flowchart. Rather than a parenthetical reference, we now include dedicated sentences that explicitly describe the exclusion of 630 children (34.5%) and direct readers to the detailed flowchart in Figure 2 of the Appendix. The key information about sample size, exclusion percentage, and rationale is now more prominently featured in the main text. |
| **Data Sharing & Transparency**   - **Data Availability**: The FigShare repository is appropriate, but a public DOI should be provided at acceptance. The current phrasing (“private link will be updated”) is not compliant with journal data policies. | We apologize that this point was not clear in our earlier response. The private link has an accompanying DOI (10.6084/m9.figshare.28711070) that will become active and public at acceptance. |
